# Abstract Versus Concrete Risk Identification in Clinical Research in Japan: Randomized and Prospective Pilot Research on the Effect of Risk Reduction Activities in a Risk-Based Approach

**DOI:** 10.1007/s43441-024-00702-w

**Published:** 2024-10-28

**Authors:** Hidenobu Kondo, Shih-Wei Chiu, Yukikazu Hayashi, Naoto Takahashi, Takuhiro Yamaguchi

**Affiliations:** 1 Division of Biostatistics, Tohoku University Graduate School of Medicine, Sendai, Japan; 2 Clinical Operation Steering Department, A2 Healthcare Corporation, Sumitomo Fudosan Korakuen Bldg. 1-4-1, Koishikawa, Bunkyo-ku, Tokyo, 112-0002 Japan; 3 Clinical Research Data Center, Tohoku University Hospital, Sendai, Japan; 4 A2 Healthcare Corporation, Tokyo, Japan; 5 Department of Hematology, Nephrology, and Rheumatology, Akita University Graduate School of Medicine, Akita, Japan

**Keywords:** Quality management system, Risk-based quality management, Risk-based approach, Risk identification, Risk evaluation, Risk reduction activities

## Abstract

**Background:**

The risk-based approach (RBA) of clinical trial was first introduced in 2011–2012. RBA necessitates implementing risk reduction activities that are proportionate to risk in order to reduce avoidable quality issues. However, there is no consistent methodology or research for identifying and evaluating risks and planning risk reduction activities. We aimed to evaluate risk reduction activities and their effects by using two risk identification and evaluation methods.

**Methods:**

Among the risk identification and evaluation methods, we selected one method with the lowest number of categories for identifying risks [risk assessment form (RAF)] and one with the highest number [risk assessment tool (RAT)]. Each method was used to identify and evaluate risks in and plan risk reduction activities for the research on ponatinib blood concentration and treatment outcome in patients with chronic phase chronic myelogenous leukemia. RAF and RAT can identify risk using abstract questions and a list of concrete risks, respectively. The sites were randomized into two groups to implement planned risk reduction activities using RAF and RAT and to compare the mean of errors and protocol deviation per subject visit between the two groups.

**Results:**

The mean of errors per subject visit and the mean of protocol deviation per subject visit were lower in the RAF group than in the RAT group.

**Conclusions:**

Our study indicates that risk reductions can be successfully implemented by using a method to identify and evaluate risks in a small number of abstract categories that are critical to quality of clinical research.

## Introduction

Clinical trials have various avoidable quality problems. While the current research practice can realize clinical trials of good quality, it can be expensive [1]. The commonly cited reason is that clinical research practices are not proportionate to risk or well adapted to achieving the desired goals [1]. The Food and Drug Administration (FDA), European Medicines Association (EMA), and Pharmaceuticals and Medical Devices Agency (PMDA) have developed guidelines for adopting the risk-based approach (RBA) [1–3] to address these issues. RBA have been also specified in the International Council for Harmonization (ICH) E6–good clinical practice (GCP) (R2) guideline for good clinical practice [4]. Moreover, the FDA, EMA, and PMDA have recommended the use of the RBA in their regulations for each country [5–7].

The ICH E6(R2) states that “risk reduction activities may be incorporated in protocol design and implementation, monitoring plans, agreements between parties defining roles and responsibilities, systematic safeguards to ensure adherence to standard operating procedures, and training in processes and procedures.” The FDA guideline does not describe risk reduction activities but only describes monitoring activities [2]. However, the EMA Reflection Paper [1], TransCelerate Position Paper [8], ECRIN [9], and Japan Pharmaceutical Manufacturers Association (JPMA) publications [10, 11] describe training as a component of risk reduction activities. RBA necessitates the implementation of risk reduction activities that are proportionate to risk in order to mitigate avoidable quality problems; that activities are adapted appropriately to achieve the desired goals. In the GCP Renovation, including ICH E8(R1) and ICH E6(R3), the focus of identifying and evaluating risks and planning risk reduction activities has shifted from the critical data and process to the critical to quality factors. Since the ICH E6(R2) was issued, various organizations such as pharmaceutical companies, academia, and contract research organizations have been investigating methods for effective risk identification and evaluation. However, no consensus has been achieved; thus, it is difficult to select appropriate methods for implementing RBA. Currently, there are 10 publicly available tools for risk identification and evaluation [9, 12–20], and 8 of them can even be used to develop risk reduction activities [9, 12–14, 16–18, 20].

The relevance of risk identification and evaluation methods and reducing the scope and frequency of on-site monitoring has been evaluated in the literature [21, 22]. However, the relevance of risk identification and evaluation methods and risk reduction activities (e.g., developing manuals, procedures, instructions, and training) has not been explored, and there is insufficient information about criteria for selecting appropriate methods. We aim to evaluate the effects of risk reduction activities by using two risk identification and evaluation methods in order to determine the most appropriate methods for planning risk reduction activities.

## Methods

### Research Information

**Figure 1:**
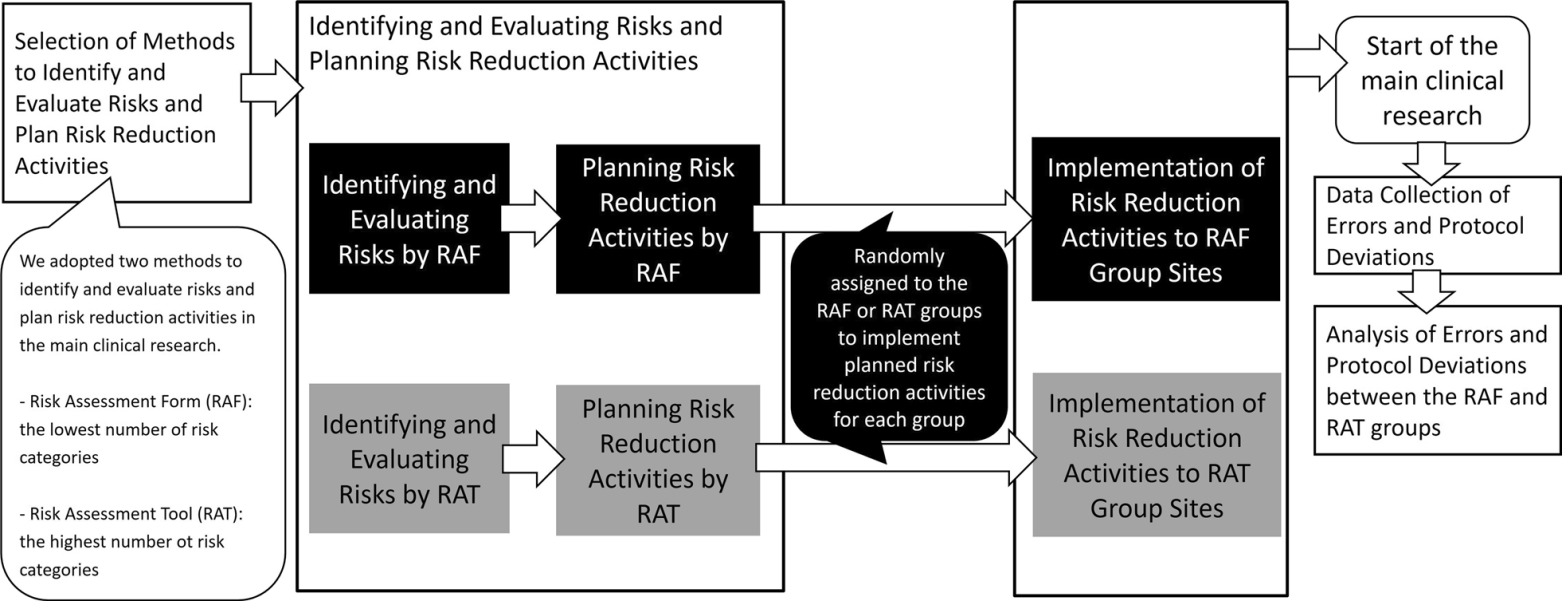
Methods Used in this Study.

The main clinical research assesses the blood concentration of ponatinib and treatment outcomes in patients with chronic phase chronic myelogenous leukemia (Clinical Research Registry Number: UMIN000035692). In this study, 17 participants at 13 sites in Japan were randomized. The primary outcome for the main clinical research is the blood concentration of ponatinib for 48 weeks in the major molecular response (MMR) achievement and the MMR nonachievement groups. Clinical data were collected using Electronic Data Capture (EDC), Viedoc. The main clinical research was chaired by Dr. Naoto Takahashi, Department of Hematology, Nephrology, and Rheumatology, Akita University Graduate School of Medicine; coordinated by the Clinical Research Innovation and Education Center, Tohoku University Hospital; and funded by Otsuka Pharmaceutical Co., Ltd. The Ethics Committee of Tohoku University Graduate School of Medicine in Miyagi, Japan approved our companion study (approval number 2022-1-877). Additional participant consent was not obtained because no personal identifiable information was used and no special risks or burdens were associated with participation in our companion study. The method used in our companion study is depicted in Fig. 1.

### Selection of Methods to Identify and Evaluate Risks and Plan Risk Reduction Activities

In addition to the systematic review of risk-based monitoring tools conducted by Caroline et al. [23], a collection of risk-based monitoring tools (https://ecrin.org/tools/risk-based-monitoring-toolbox) in ECRIN was employed to collect publicly available methods of risk identification and evaluation. In addition, the Pubmed database was searched for articles on newly reported methods since the study conducted by Caroline et al. [23]. We summarized the obtained information on risk identification and evaluation methods, including the intended use of the results of risk identification and evaluation, the type of risk identification, the number of risk categories that are most detailed in each method, and the type of risk evaluation, as shown in Table 1. Two typical types of risk identification are commonly used in RBA: identifying risks using abstract categories and selecting applicable risks from a list of concrete risks. In this study, we selected two methodologies that contradict each other in terms of risk identification type and the number of risk categories, based on the information summarized in Table 1 because these two types are widely used in clinical trials for risk identification. We believed that these contradicting methods would yield obviously different results. One of these methods, the risk assessment form (RAF) [17], had the lowest number of risk categories that are most detailed; in contrast, the risk assessment tool (RAT) [16] had the highest number of risk categories that are most detailed. The number of the most detailed categories in RAF and RAT is 12 and 164, respectively. The main categories of RAF are “Patient Hazards/Research Staff Hazards (Rights and Safety)”, “Study Hazards (Completion and Reliability)” and “Organizational Hazards”. RAF was used to identify risks in 12 abstract categories. The main categories of RAT are “Study organisation and governance”, “Training”, “Trial subjects rights and safety”, “Data”, “Protocol procedures”, “Study Drug/IMP”, “Safety management”, “Impact” and “Other”. RAT was used to select relevant risks from a list of 164 concrete risks. Among the categories in RAF, “Liability” and “Intellectual Property” in “Organizational Hazards” did not have any equivalents in the RAT categories. As RAF scores “Impact” and “Likelihood” on a five-point scale and RAT scores “Severity” and”Probability” on a three-point scale, the two risk evaluation methods are similar, although their number of evaluation points is different.

**Table 1:** Summary of the Risk Identification and Evaluation Methods.

Author, Published Year	Tool Name/Research Name	Intended Use of the Results of Risk Identification and Evaluation				Type of Risk Identification	Number of Risk Categories that are the Most Detailed	Type of Risk Evaluation
		Plan Risk Reduction Activities	Calculation of Overall Risk Level of Research	Decision of Monitoring Intensity	Decision of Monitoring Method			
TransCelerate, 2014 [12]	Risk assessment and categorization tool	X	X	X	X	Users identify risks concretely (table style)	118	Impact (3 levels), Probability (3 levels), Detectability (3 levels), Weighting
MRC/DH/MHRA Joint Project, 2011 [13]	Guidance on risk-proportionate approaches to the management and monitoring of clinical trials	X		X	X	Users identify risks concretely (table style)	61	No settings
ADAMON, 2009 [14]	Risk analysis in clinical trials regarding the required amount of on-site monitoring	X		X		Users identify risks concretely (no table)	23	Options along the flow
OPTIMON, 2008 [15]	Optimon grid for patient risk evaluation in academic clinical research studies			X	X	No settings	No settings	No settings
Nordic Monitoring Network, 2015 [16]	Risk assessment tool (RAT)	X			X	Users identify risks selectively	164	Probability (free text or 3 levels), Severity (free text or 3 levels)
Smith, 2014 [17]	Risk assessment form (RAF)	X	X		X	Users identify risks concretely (table style)	12	Impact (5 levels), Likelihood (5 levels)
Swiss Clinical Trial Organisation, 2015 [18]	Risk assessment for risk-adapted monitoring	X		X	X	Users identify risks selectively	23	Multiple choice
University of Minnesota Masonic Cancer Center, 2017 [19]	Masonic Cancer Centre (MCC) risk assessment checklist			X		No settings	No settings	No settings
ECRIN, 2008 [9]	ECRIN guidance document on risk assessments	X		X	X	No settings	19	No settings
F-CRIN/ECRIN, 2015 [20]	Guideline on risk management for clinical research	X				Users identify risks concretely (table style)	18	Likelihood (10 levels), Detectability (10 levels), Gravity (or severity) (10 levels)

### Identifying and Evaluating Risks and Planning Risk Reduction Activities

We used the RAF and RAT methods to identify and evaluate risk and plan risk reduction activities from October 3, 2018 to December 5, 2018. Risk identification and evaluation and planning of risk reduction activities were first performed with RAF and then with RAT. The activities were performed by four data managers (DMs), two clinical research associates (CRAs), and two statisticians from the Clinical Research Data Center of the Clinical Research Innovation and Education Center at Tohoku University Hospital. An RBA specialist with experience in identifying and evaluating risks and planning risk reduction activities in more than 10 clinical trials was the facilitator. The facilitator was required to participate in the study at the time of implementation, but the participation of other members was voluntary, with at least four members participating. One facilitator and one DM had experience in identifying and evaluating risks and planning risk reduction activities in clinical research. For these activities, we did not differentiate between RAF and RAT participants. The risk reduction activities involved communicating precautions and conducting training in processes and procedures to site staffs and setting alert texts to appear on the EDC screen. The alert texts were used to prevent mistakes or protocol deviations, not as queries to correct data entry errors.

### Deciding on the Sites to Implement Risk Reduction Activities

The sites participating in the main clinical research were randomly assigned to the RAF or RAT groups to implement planned risk reduction activities for each group. Randomization and stratification were performed by the CRAs (n = 3) to ensure approximate balance between RAF and RAT (1:1) and avoid the biases caused by CRA. The RAF and RAT groups were equally likely to appear. The total number of participating sites was 13, and the number of CRAs was 3; thus, the CRAs oversaw five, five, and three sites. The RANUNI function of the SAS System Release 9.4 software (SAS Institute Japan Ltd., Tokyo, Japan) was used for random number generation. After the randomization, six sites were assigned to the RAF group and seven to the RAT group. However, after the assignment, the number of participating sites in the main clinical research decreased to five in the RAF group and seven in the RAT group (Table 2).

**Table 2:** Characteristics by Randomization Group.

	RAF Group	RAT Group
Number of sites	5	7
Person-time required to implement risk identification and risk evaluation and plan risk reduction activities (person-hours)	33.18	35.59
Number of identified risks	21	41
Total number of risk reduction activities	12	16
Communicating precautions	7	7
Training in processes and procedures	4	5
Setting of alert texts to appear on the EDC screen	1	4
Number of sites with participant enrollment	5	5
Number of subjects	6	11
Number of subject visits	75	114

### Implementation of Risk Reduction Activities

We prepared separate Microsoft PowerPoint files containing content explaining the risk reduction activities—which involved communicating precautions and training in processes and procedures—for the RAF and RAT groups. An audio version of the content was prepared, and two separate videos were created for the RAF and RAT groups. The risk reduction activities were performed by this video with the investigators and the site staff of every site on the CRA’s first visit. In addition, the slides were distributed to the principal investigator and site staff at the time of the CRA’s visit. As part of the risk reduction activities, each alert text appearing on the EDC screen was described by the RAF and RAT groups. All investigators were trained with regard to the content of the risk reduction activities during the investigator meeting prior to the initiation of the main clinical research. In the risk reduction activities, precautions and training were additionally conveyed in video format and an alert text was set to appear on the EDC screen in the RAF group for the risk of missing of ponatinib blood concentration measurement. In the RAT group, an additional risk reduction activity was implemented, in which the alert text was set to appear on the EDC screen for the risk of inadequate and lack of SAE registration and reporting. The deadline for data entry and the need for entering protocol deviation information into EDC are described in the case report form (CRF) completion guideline. To ensure the common risk reductions in the main clinical research, the deadline for data entry and the need for entering protocol deviation information were shared with both groups. In the RAF group, the additional risk reduction activities were implemented, in which the precautions and training of the deadline for data entry were conveyed in video format. In the RAT group, an additional risk reduction activity was implemented, in which the precautions and training of entering protocol deviation information were conveyed in video format.

### Data Collection of Errors and Protocol Deviations

The implementers, implementation date, start time, and end time of the risk identification, risk evaluation, and planning of the risk reduction activities were recorded and collected.

On-site, off-site, and central monitoring were conducted in this study. On-site monitoring was conducted using partial source data verification (SDV) and source data review (SDR), targeting informed consent, eligibility of the study participants, and source documents and data up to the 4-week visit. In case of problems in on-site monitoring up to the 4-week visit, on-site monitoring was conducted after 24 weeks and after discontinuation or termination. If there were no problems for up to 4 weeks, off-site monitoring was conducted after the 4-week visit. On-site or off-site monitoring was conducted in cases of serious adverse events (SAEs).

The CRAs queried data that required correction of the electronic CRF (eCRF) and the source data during on-site monitoring. The data queried by the CRAs were used as error data. DMs also created queries after the data check. The data queried by DMs were used as error data only when the data were updated after the query date. The protocol deviation information was obtained by extracting the protocol deviation dataset from the EDC.

### Analysis of Errors and Protocol Deviations

We calculated point estimates and 95% confidence intervals (CI) for means and differences of mean between the RAF and RAT groups for the following items:

- Amount of error data per subject visit
- Number of protocol deviations per subject visit
- Amount of error data per person-time required to implement risk identification and evaluation and plan risk reduction activities
- Number of protocol deviations per person-time required to implement risk identification and evaluation and plan risk reduction activities

The amount of error data was also compared between the data risk categories, which were determined using the impact of data errors, classified as high, medium, or low. The high-risk category included critical data related to the protection of human participants, eligibility criteria, informed consent, and primary endpoints. The medium-risk category included important data not related to the protection of human participants, eligibility criteria, informed consent, and primary endpoints. The low-risk category included all other data not covered by the previous two categories. Because all data were classified into high or medium risk categories, none of the data were classified in the low-risk category.

## Results

The person-time for risk identification, risk evaluation, and planning risk reduction activities using RAF and RAT were 33.18 person-hours and 35.59 person-hours, respectively (Table 2). The number of risks identified using RAF and RAT, including those withheld at the time of initial risk identification owing to missing details, was 21 and 41, respectively (Table 2). The total number of risk reduction activities was 12 in RAF: communicating precautions (7), conducting training in processes and procedures (4, including the distribution of materials), and setting alert texts to appear on the EDC screen (1). The total number of risk reduction activities was 16 in RAT: communicating precautions (7), conducting training in processes and procedures (5, including the distribution of materials), and setting alert texts to appear on the EDC screen (4) (Table 2). The number of sites with participants enrolled in both groups was five (Table 2).

The means of error data per subject visit for the medium- and high-risk categories were not significantly different in the RAF and RAT groups (Table 3). The differences in the means of error data per subject visit between the two groups were 0.54 (95% CI  − 0.760 to 1.836) for the medium-risk categories and 0.35 (95% CI − 0.805 to 1.497) for the high-risk categories (Table 3). There were small differences in the data for the high-risk category, with values of 0.70 /visit (95% CI 0.296 to 1.104) for the RAF group and 1.05/visit (95% CI 0 to 2.216) for the RAT group (Table 3). There were large differences in the data for the medium-risk category, with values of 0.38 /visit (95% CI 0.248 to 0.520) for the RAF group and 0.92 /visit (95% CI 0 to 2.224) for the RAT group (Table 3).

**Table 3:** Analysis of the Amount of Error Data Per Subject Visit for Data Risk Categories in the RAF and RAT Groups and Number of Protocol Deviations Per Subject Visit in These Groups.

		RAF Group	RAT Group	Difference (RAT–RAF)
Number of sites		5	5	
Amount of error data for medium-risk category per subject visit (n/visit)	Mean	0.38	0.92	0.54
	95% CI	(0.248, 0.520)	(0, 2.224)	(− 0.760, 1.836)
Amount of error data for high-risk category per subject visit (n/visit)	Mean	0.70	1.05	0.35
	95% CI	(0.296, 1.104)	(0, 2.216)	(− 0.805, 1.497)
Number of protocol deviations per subject visit (n/visit)	Mean	0.37	0.58	0.21
	95% CI	(0, 0.773)	(0.151, 1.013)	(− 0.277, 0.705)

The mean of protocol deviations per subject visit was not significantly different in the RAF and RAT groups (Table 3). There were 24 and 42 deviations in the RAF and RAT groups, respectively. The means of error data and protocol deviations per person-time required to implement risk identification and evaluation and plan risk reduction activities were not significantly different in the RAF and RAT groups (Table 4). The mean of error data per person-time required to implement risk identification and evaluation and plan risk reduction activities was 0.47 /person-hour (95% CI 0.263 to 0.673) in the RAF group and 0.96/person-hour (95% CI 0 to 2.235) in the RAT group (Table 4). The result per subject visit was 0.03/person-hour/visit (95% CI 0.019 to 0.046) for the RAF group and 0.06/person-hour/visit (95% CI 0.0000 to 0.111) for the RAT group (Table 4). The number of protocol deviations per person-time required to implement risk identification and evaluation and plan risk reduction activities was 0.15/person-hour (95% CI 0 to 0.303) in the RAF group and 0.24/person-hour (95% CI 0.132 to 0.340) in the RAT group (Table 4). The result per subject visit was 0.01/person-hour/visit (95% CI 0 to 0.024) for the RAF group and 0.02/person-hour/visit (95% CI 0.004 to 0.029) for the RAT group (Table 4).

**Table 4:** Analysis of the Number of Error Data and Protocol Deviations Per Person-Time to Implement Risk Identification and Risk Evaluation and Plan Risk Reduction Activities in the RAF and RAT Groups.

		RAF Group	RAT Group	Difference (RAT − RAF)
Person-time required to implement risk identification and risk evaluation and plan risk reduction activities (person-hours)		33.18	35.59	
Amount of error data per person-time required to implement risk identification and risk evaluation and plan risk reduction activities (n/person-hour)	Mean	0.47	0.96	0.49
	95% CI	(0.263, 0.673)	(0, 2.235)	(− 0.782, 1.758)
Amount of error data per person-time required to implement risk identification and risk evaluation and plan risk reduction activities and per subject visit (n/person-hour/visit)	Mean	0.03	0.06	0.02
	95% CI	(0.019, 0.046)	(0.000, 0.111)	(− 0.032, 0.078)
Number of protocol deviations per person-time required to implement risk identification and risk evaluation and plan risk reduction activities (n/person-hour)	Mean	0.15	0.24	0.09
	95% CI	(0, 0.303)	(0.132, 0.340)	(− 0.071, 0.242)
Number of protocol deviations per person-time required to implement risk identification and risk evaluation and plan risk reduction activities and per subject visit (n/person-hour/visit)	Mean	0.01	0.02	0.01
	95% CI	(0, 0.024)	(0.004, 0.029)	(− 0.010, 0.020)

## Discussion

This study aims to facilitate the selection of an appropriate method for identifying and evaluating risks and planning risk reduction activities.

The mean of error data per subject visit, mean of protocol deviations per subject visit, and actual number of protocol deviations were lower in the RAF group than in the RAT group. We identified fewer risks using RAF than RAT. These results may imply that the RAF method is effective in risk identification and evaluation and risk mitigation planning by identifying specific risks in a small number of abstract categories. While the means of error data and protocol deviations per subject visit were not statistically significantly different in the RAF and RAT groups possibly owing to the small sample size, the number of errors in the medium-risk category in the RAT group was greater than twice that of the RAF group. This implies that there is no difference between the two methods in terms of ensuring reliability of the trial results.

The means of error data and protocol deviations per person-time required to implement risk identification and evaluation and plan risk reduction activities were both lower in the RAF group than in the RAT group. These results may be attributable to differences in the risk identification method whether it is abstract or concrete risk identification. As no risk reduction activity was identified in the categories which are different between the two methods, the difference in the risk categories might not affect the results. In addition, as the number of evaluation points might not affect the determination of the content of the risk reduction activities, this difference in the risk evaluation method might also not affect the results.

DMs, CRAs, statisticians from the Clinical Research Data Center of the Clinical Research Innovation and Education Center at Tohoku University Hospital, and RBA specialists conducted risk identification and evaluation and planed risk reduction activities in this study. Unlike the researchers, individuals who are unfamiliar with clinical research or those who have no experience in risk identification and evaluation and planning risk reduction activities should be given more time for identifying and evaluating risks and planning risk reduction activities.

These knowledge of clinical research and experience in risk identification, risk evaluation, and planning of risk reduction activities might have a significant impact on RAF as it is used to identify risks from 12 abstract categories, and the derivation of risks from abstract questions requires such knowledge and experience. Therefore, risk identification from abstract categories or questions should be employed by those who are familiar with clinical research or who have experience in risk identification, risk evaluation, and risk reduction activities. Otherwise, RAT may be a more suitable method.

In RAF group, the additional risk reduction activity for the risk of missing data entry was implemented. However, the total number of missing data entry per total subject visits was lower in the RAT group (RAF group: 0.25/visit, RAT group: 0.07/visit, data not shown). Similarly, the additional risk reduction activity for the risk of missing data entry of protocol deviation was implemented in RAT group and the total number of missing data entries of protocol deviation per total subject visits was lower in the RAF group (RAF group: 0.03/visit, RAT group: 0.07/visit, data not shown). These results may be attributable to the difficulty of consciously maintaining the precaution of data entry as the timeliness of data entry is generally trained in clinical research and is not a clinical research-specific precaution for investigators. In addition, although no entry of protocol deviation was considered a clinical research–specific risk, it may have been perceived by the site staff as common in terms of data entry. The problem of missing data entry may not be effectively addressed through risk reduction activities such as the communication of precautions and training.

In RAF group, the additional risk reduction activity for the risk of missing of ponatinib blood concentration measurement was implemented. The total number of deviations per total subject visits related to this measurement was lower in the RAF group (RAF group: 0.01/visit, RAT group: 0.04/visit, data not shown). The risk reduction activity communicating SAE reporting procedures and the deadline for SAE data entry was implemented in both groups, and the precautions and training were conveyed in video format. Similarly, the additional risk reduction activities for SAE reporting were implemented in RAT group and the total number of deviations per total subject visits related to this SAE reporting was lower in the RAT group (RAF group: 0.03/visit, RAT group: 0.01/visit, data not shown). All investigators received training regarding the contents of the risk reduction activities during the investigator meeting prior to the main clinical study. Therefore, one-way communication methods, such as watching videos for precautions and training, and presenting alert texts on the EDC screen, may effectively reduce the risks.

Our study indicates that protocol deviations can be prevented by setting the alert text to appear on the EDC screen, in addition to training, which is commonly used as a risk-mitigating measure in the EMA Reflection Paper [1], TransCelerate Position Paper [8], ECRIN [9], and JPMA publications [10, 11]. In clinical research in which CRAs find it difficult to mitigate risks or take preventive actions, setting the alert text to appear on the EDC screen may provide low-cost quality control. In clinical trials conducted by pharmaceutical companies, the CRF design has become more standardized, and such efforts have not been implemented, although they may be effective for critical data.

A limitation of our study is that the main clinical research was conducted with a small sample size. Therefore, the results cannot be generalized. In addition, risk identification, risk evaluation, and the planning of risk reduction activities were performed by the same researchers. Planning activities using RAF before using RAT may have affected the results.

Because 100% SDV and SDR were not conducted in the main clinical research, not all errors and protocol deviations may have been identified. However, the scope of monitoring and data review covered all data, thus ensuring the comparability of RAF and RAT.

Our study indicates that RBA can be effectively used to identify risks in a small number of abstract categories. Risk identification, risk evaluation, and the planning of risk reduction activities are commonly practiced in clinical trials. However, to the best of our knowledge, this is the first study to evaluate the relationship between risk identification and evaluation and risk reduction activities. Critical to quality factors of clinical trials can be considered abstract categories; thus, this research result may help ensure adherence to the policy of risk identification outlined in the GCP Renovation.

## Conclusion

Our study indicates that risk reduction activities can be successfully implemented by using an appropriate method to identify and evaluate risks in a small number of abstract categories that are critical to the quality of clinical research. The results can facilitate selection of an appropriate risk identification and evaluation method for planning risk reduction activities.

## Data Availability

No datasets were generated or analysed during the current study.
